# Antigen exposure required for T cell activation

**DOI:** 10.1186/2045-7022-4-S3-P115

**Published:** 2014-07-18

**Authors:** Xiaoli Meng, Fiazia Yaseen, Caroline Earnshaw, Roz Jenkins, Daniel Peckham, Paul Whitaker, Neil French, Munir Pirmohamed, Dean Naisbitt, Kevin Park

**Affiliations:** 1 Liverpool University, MRC Centre for Drug Safety Science, UK; 2 Regional Adult Cystic Fibrosis Unit., St. James’s Hospital, UK

## Background

A high frequency of hypersensitivity reactions to -lactam antibiotics are observed in patients with cystic fibrosis. -lactam antibiotics form protein conjugates in vitro and in vivo, and a core group of lysine residues of human serum albumin have been shown to be penicilloylated by mass spectrometric methods. Furthermore, protein/peptide conjugates have been shown to stimulate T cells isolated from patients with -lactam hypersensitivity. However, the threshold level of protein conjugation required to trigger immune responses has not been studied.

## Method

Thus, we focused on piperacillin, a commonly used drug in patients with cystic fibrosis, to (a) quantify the piperacillin antigens formed in patients using mass spectrometry and a synthetic piperacillin-modified albumin peptide (539ATK(Pip)EQLK545) as a standard and (b) determine the quantity of the piperacillin protein adducts formed at the time of T cell activation. Plasma was collected prior to commencing treatment and after a 14 day treatment course from 10 patients and the level of piperacillin-modified albumin in patient plasma was measured. Piperacillin-specific CD4+ T-cell clones were generated from hypersensitive patients by serial dilution and cultured with soluble drug or antigen presenting cells pulsed with piperacillin for 1-48h for the analysis of drug-specific proliferative responses. At each time-point piperacillin albumin binding was quantified.

## Results

Piperacillin-modified lysine was detected in human serum albumin in all 10 patients; the level of piperacillin-modified lysine 541 was found to range from 2.6 to 6.5%. Antigen presenting cells pulsed with piperacillin for 1 and 4h did not stimulate a strong T-cell proliferative response and this coincided with low levels of albumin modification. In contrast, antigen presenting cells pulsed with piperacillin for 24 or 48h activated all of the clones. Quantitative analysis of incubation medium revealed that approximately 3% of lysine 541 was modified after 24h.

## Conclusion

In conclusion, these data quantify for the first time, the level of piperacillin albumin binding in drug exposed patients and in vitro at drug antigen concentrations that activate piperacillin-specific T-cells.

